# Exploring complex discourse to index executive functioning in older adults with cognitive impairment

**DOI:** 10.1093/geroni/igaf122.4333

**Published:** 2025-12-31

**Authors:** Eliza Baby, Lizzy Lydon, Natalia Rzepa, Sara Czaja, Walter Boot, Neil Charness, Wendy Rogers, Raksha Mudar

**Affiliations:** University of Illinois at Urbana-Champaign, Champaign, Illinois, United States; Miami University, Oxford, Ohio, United States; University of Illinois Urbana-Champaign, Urbana-Champaign, Illinois, United States; Weill Cornell Medicine, New York, New York, United States; Weill Cornell Medicine, New York, New York, United States; Weill Cornell Medicine, New York, New York, United States; University of Illinois Urbana-Champaign, Champaign, Illinois, United States; University of Illinois Urbana-Champaign, University of Illinois Urbana-Champaign, Illinois, United States

## Abstract

Discourse production engages multiple cognitive domains including executive functions and is known to be disrupted in individuals with cognitive impairments. Studying discourse reveals how cognitive impairments affect daily functioning by highlighting challenges in communication. We evaluated utility of a complex discourse production task in characterizing differences across older adults (n = 90) with mild cognitive impairment (MCI), traumatic brain injury (TBI), post-stroke cognitive impairment (PSCI), multiple etiology cognitive impairment, and cognitively normal older adult controls as part of the Everyday Needs Assessment for Cognitive Tasks (ENACT) study. Using a mixed-methods, cross-sectional design (n = 18 per group; mean age=71.81 ± 3.36 years) participants completed a discourse production task requiring executive processes (e.g., abstraction, planning) along with standard cognitive assessments. Discourse was analyzed using manual and automated methods. Manual coding identified critical elements (travel, activity, packing, and stay) and elaborations within categories. Automated analysis via CLAN software extracted linguistic metrics such as mean length of utterance (MLU) and semantic density. Group differences were analyzed using ANCOVA with age as a covariate. Significant group differences (p < 0.05), controlling for multiple comparisons, emerged across several discourse measures. The MCI and multiple etiology groups produced fewer critical elements compared to controls, whereas all other clinical groups showed reduced elaborations and semantic density. Additionally, participants in the TBI and PSCI groups had shorter utterances reflected in the lower MLU scores. Findings suggest that the discourse task distinguishes group-level differences and offers clinically relevant insights to guide assessment of functional challenges and inform targeted cognitive rehabilitation strategies including technology support.

